# Microbially Mediated Hydrogen Cycling in Deep-Sea Hydrothermal Vents

**DOI:** 10.3389/fmicb.2018.02873

**Published:** 2018-11-23

**Authors:** Nicole Adam, Mirjam Perner

**Affiliations:** Geomicrobiology, GEOMAR Helmholtz Centre for Ocean Research Kiel, Kiel, Germany

**Keywords:** hydrogen cycling, hydrogen consumption, hydrogenases, hydrogen oxidizers, hydrothermal vent

## Abstract

Deep-sea hydrothermal vents may provide one of the largest reservoirs on Earth for hydrogen-oxidizing microorganisms. Depending on the type of geological setting, hydrothermal environments can be considerably enriched in hydrogen (up to millimolar concentrations). As hot, reduced hydrothermal fluids ascend to the seafloor they mix with entrained cold, oxygenated seawater, forming thermal and chemical gradients along their fluid pathways. Consequently, in these thermally and chemically dynamic habitats biochemically distinct hydrogenases (adapted to various temperature regimes, oxygen and hydrogen concentrations) from physiologically and phylogenetically diverse Bacteria and Archaea can be expected. Hydrogen oxidation is one of the important inorganic energy sources in these habitats, capable of providing relatively large amounts of energy (237 kJ/mol H_2_) for driving ATP synthesis and autotrophic CO_2_ fixation. Therefore, hydrogen-oxidizing organisms play a key role in deep-sea hydrothermal vent ecosystems as they can be considerably involved in light-independent primary biomass production. So far, the specific role of hydrogen-utilizing microorganisms in deep-sea hydrothermal ecosystems has been investigated by isolating hydrogen-oxidizers, measuring hydrogen consumption (*ex situ*), studying hydrogenase gene distribution and more recently by analyzing metatranscriptomic and metaproteomic data. Here we summarize this available knowledge and discuss the advent of new techniques for the identification of novel hydrogen-uptake and -evolving enzymes from hydrothermal vent microorganisms.

## Introduction

Hydrogen conversion, the reversible reaction of molecular hydrogen (H_2_) to protons and electrons, plays a major role for metabolic processes in microbial cells: generally, energy conservation and the recycling of reducing equivalents (in microbial fermentation or light-dependent photosynthesis) is accomplished by enzymatic hydrogen evolution (Vignais and Billoud, 2007; Hallenbeck, 2009). Enzymatically catalyzed hydrogen oxidation is widely distributed among prokaryotes, and can power the synthesis of energy-rich ATP, which is needed for autotrophic carbon fixation (Dilling and Cypionka, 1990; Bothe et al., 2010; Greening et al., 2016).

**FIGURE 1:**
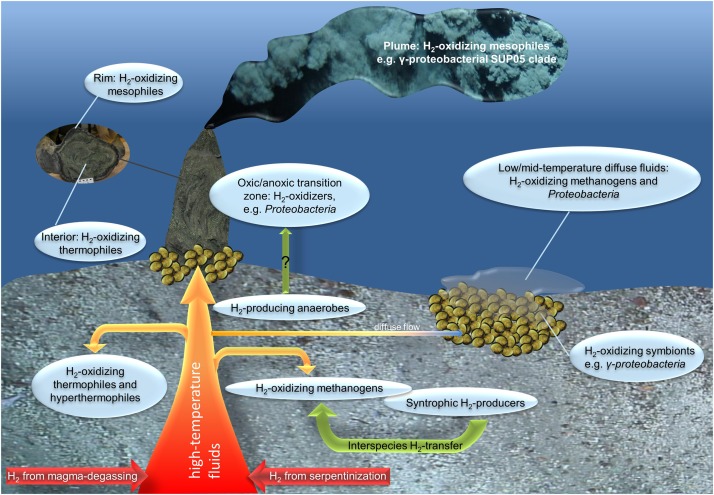
Overview of possible (microbially mediated) hydrogen cycling in hydrothermal vent systems. Hydrothermal emissions emanate from the subsurface either as high-temperature focused fluids causing the typical black or white smoker structures or low- to mid-temperature diffuse fluids (for example from mussel beds). Abiogenic hydrogen sources are displayed in red and orange (hydrothermal fluids) and biogenic hydrogen sources in the shape of green arrows.

The thermal (4°C to several 100s °C) and chemical (e.g., oxidized to reduced) gradients hallmarking deep-sea hydrothermal vent habitats have the potential to host one of the largest reservoirs of physiologically and phylogenetically diverse hydrogen-converting microorganisms (Figure 1, Kelley et al., 2002; Perner et al., 2013b). As the fluids pass through the subsurface, they get enriched in various inorganic compounds, such as reduced minerals, sulfide and hydrogen (Figure 1). The actual hydrogen and sulfide concentrations of the emanating fluids strongly depend on the type of host rock underlying the respective vent system and the mixing ratio of seawater and fluids. Hydrothermal end-member fluids of basalt-hosted systems are usually characterized by greater sulfide than hydrogen concentrations, resulting from magma degassing and high-temperature-leaching from enclosing host rocks. In contrast, due to serpentinization processes, end-member fluids of ultramafic-hosted vent systems usually exhibit greater hydrogen (up to 1–10 M) and methane (on mM levels) concentrations than sulfide concentrations (Charlou et al., 2002; Kelley et al., 2005; Haase et al., 2007; Perner et al., 2013b). Correspondingly, sulfide oxidation in the sulfide-rich basalt-hosted and hydrogen oxidation and methanotrophy in the hydrogen-rich ultramafic-hosted systems are estimated to be the predominant sources of metabolic energy available in venting habitats (McCollom, 2007).

Since the discovery of hydrothermal vents in the late 70s (Corliss et al., 1979), numerous hydrogen-oxidizers have been isolated from thermally and chemically distinct deep-sea vent habitats (e.g., Campbell et al., 2006; Miroshnichenko and Bonch-Osmolovskaya, 2006; Nakagawa and Takai, 2008; Hansen and Perner, 2015; Nagata et al., 2017). Although considerable efforts have been undertaken to promote our understanding of the distribution and role of hydrogen-oxidizing organisms in these environments (Nealson et al., 2005; Campbell et al., 2006; Perner et al., 2010, 2013b; Petersen et al., 2011; Adam and Perner, 2018), our knowledge of the overall hydrogen utilization potential and microbial hydrogen pathways still remains limited. This review summarizes the work that has been done on hydrogen-metabolizing microorganisms colonizing hydrothermally influenced environments with respect to their diversity, hydrogen consumption rates in incubation experiments and protein biochemistry. Recent findings in the context of culture-independent metagenomic and metatranscriptomic approaches for the identification of novel hydrogen-converting enzymes are included. Finally, an outlook is given which techniques (e.g., *in situ* experiments) and work are needed to advance our understanding of the role that hydrogen-cycling microorganisms play in hydrothermal vents.

## Hydrogen-Producing and -Oxidizing Microorganisms

It is well known that hydrogen-producing and -oxidizing microorganisms can coexist or even interact in a variety of anoxic habitats like sediments or intestinal tracts (Chassard and Bernalier-Donadille, 2006). At low hydrogen partial pressures (e.g., <100 Pa), hydrogen can be produced in the course of microbial fermentation processes (Kraemer, 2007; Hallenbeck, 2009) which is then oxidized by hydrogenotrophic microorganisms, especially methanogens. This interspecies hydrogen transfer thereby forms so-called syntrophic communities (hydrogen-producers and –consumers thrive in close proximity) and most likely represents an important hydrogen source in hydrogen-poor habitats (Bryant et al., 1977; Chassard and Bernalier-Donadille, 2006). Since fermentative hydrogen production can already be inhibited at relatively low hydrogen concentrations (i.e., on a nM level) (Wolin, 1976; Hoehler et al., 1998; Hallenbeck, 2009), the role, that microbially produced hydrogen plays in hydrothermal vent systems, remains enigmatic. Even the hydrogen levels of hydrogen-poor hydrothermal vent systems easily exceed those of habitats known to harbor fermentative bacteria like sediments (which are typically below 60 nM) (Novelli et al., 1987; Hoehler et al., 1998; Charlou et al., 2002; Perner et al., 2013b) and thus are likely above the inhibitory limit for biological hydrogen production. This may explain why studies on microbial hydrogen production in deep-sea hydrothermal vent systems have been largely neglected so far. However, hydrogen-evolving heterotrophic Archaea and Bacteria have been identified in hydrothermal fluid incubation experiments (Topcuoglu et al., 2016). The authors posited that in some of the micro niches represented by the culturing conditions, hyperthermophilic *Euryarchaeota* and thermophilic *Firmicutes* produced hydrogen as a waste product during fermentation which was consumed by hydrogenotrophic sulfate-reducing Bacteria or methanogenic Archaea (under distinct temperature regimes) (Topcuoglu et al., 2016). Hydrogenotrophic methanogens can use hydrogen to reduce CO_2_ via the reductive acetyl-CoA pathway (Wood-Ljungdahl pathway), thereby forming methane (Ladapo and Whitman, 1990; Thauer, 1998). Acetogenic Bacteria (producing acetate from CO_2_) can compete with hydrogenotrophic methanogens in anoxic, hydrogen-rich habitats using the same electron donor (hydrogen) and carbon fixation pathway (Wood Ljungdahl pathway) (Chassard and Bernalier-Donadille, 2006). Due to a lower hydrogen threshold (minimum hydrogen concentration required for hydrogenotrophic growth) and a greater overall energy yield from the conversion of CO_2_ to methane, methanogenic Archaea are usually the dominating group in this competition (Ragsdale and Pierce, 2008 and references therein). Moreover, acetogens (and methanogens) can be outcompeted by Bacteria with an even lower hydrogen threshold than methanogens, such as *Campylobacterota*, which are highly abundant at hydrothermal vent sites and take advantage of their versatile metabolisms (for details see below). Therefore, active acetogenic Bacteria are presumably less abundant in venting biotopes and have so far not been the focus of research related to hydrogen utilization in deep-sea hydrothermal vent environments.

Overall, sulfide and thiosulfate oxidation as well as hydrogen oxidation are among the chemosynthetic reactions which provide the greatest energy yields in hydrothermal vent biotopes (Amend and Shock, 2001; Fuchs et al., 2007). Although considerably more energy is yielded through oxidation of sulfide or thiosulfate than through hydrogen oxidation (free standard enthalpies are -797 kJ/mol H_2_S vs. -237 kJ/mol H_2_ with O_2_ as electron acceptor) (Table 1, Fuchs et al., 2007), the latter reaction is favorable for autotrophic carbon fixation. Since the redox-potential of hydrogen is more negative than that of the reducing equivalent NAD(P)/H, in contrast to sulfide, a reverse electron transport is not required in conjunction with hydrogen oxidation. Thus, only a third of the energy is required for fixing 1 mol of carbon when oxidizing hydrogen compared to sulfide (1060 kJ for hydrogen vs. 3500 kJ for sulfide) (Heijnen and Van Dijken, 1992). The individual fluid compositions of different hydrothermal systems may even increase this effect: depending on hydrogen and sulfide concentrations as well as other abiotic factors, such as temperature and pressure, thermodynamic models for fluids of ultramafic vent fields predict that between 10 to 18 times more energy per kg of fluid can be yielded by hydrogen oxidation compared to sulfide oxidation (McCollom and Shock, 1997; McCollom, 2007; Petersen et al., 2011). The actual energy yields of the respective oxidation reactions strongly depend on the type of terminal electron acceptor used in the metabolism, where coupled to oxygen reduction the greatest energy amount is gained (Table 1, Conrad, 1996). Alternative electron acceptors commonly used by hydrogen-oxidizing microorganisms are sulfate, Fe (III) and nitrate (Vignais and Billoud, 2007), but also elemental sulfur and CO_2_ as well as different metals, e.g., Mn (III/IV), U (VI), Cr (VI), Co (III) and Tc (VII), can be reduced by hydrogen-consumers (Table 1, Liu et al., 2002; Nakagawa and Takai, 2008). Due to mixing processes with oxygenated, ambient seawater, deep-sea hydrothermal fluids may contain numerous possible electron acceptors (primarily oxygen, nitrate, sulfate, elemental sulfur and iron). Their individual concentrations may vary strongly, depending on the geological setting of the vent system and the seawater mixing ratio.

**Table 1:** Overall reactions and standard free reaction enthalpies of hydrogen oxidation coupled to different electron acceptors.

Reaction	ΔG^′0^	Reference
2 H_2_ + **O_2_**→ 2 H_2_O	-297 kJ/mol H_2_	Fuchs et al., 2007
5 H_2_ + 2 **NO_3_^-^** + 2 H^+^ → N_2_ + 6 H_2_O	-224.2 kJ/mol H_2_	
H_2_ + **MnO_2_** → Mn^2+^ + 2 OH^-^	-166 kJ/mol H_2_	Konhauser, 2006
0.5 H_2_ + **Fe(OH)_3_** → Fe^2+^ + 2 OH^-^ + H_2_O	-110 kJ/mol H_2_	
H_2_ + **(2/3)CrO_4_^2-^** + (4/3)H^+^ → (2/3)Cr(OH)_3_ + (2/3) H_2_O	-98.35 kJ/mol H_2_	Liu et al., 2002
H_2_ + **UO_2_^2+^** → 2H_+_ + UO_2_	-92 kJ/mol H_2_	Konhauser, 2006
H_2_ + 2 **Co(III)EDTA**^-^ → 2 Co(II)EDTA^2-^+ 2 H^+^	-68.5 kJ/mol H_2_	Liu et al., 2002
H_2_ + (2/3)**TcO4**^-^ → (2/3)TcO2 + (4/3)H_2_O	-66.99 kJ/mol H_2_	
4 H_2_ + **SO4^2^**^-^ → H_2_S + 2 OH^-^ +2 H_2_O	-38 kJ/mol H_2_	Konhauser, 2006
4 H_2_ + **CO_2_** → CH_4_ + H_2_O	-32.75 kJ/mol H_2_	Fuchs et al., 2007
H_2_ + **S^0^** → H_2_S	-28 kJ/mol H_2_	Konhauser, 2006


*Electron acceptors are indicated by bold letters.*

**FIGURE 2:**
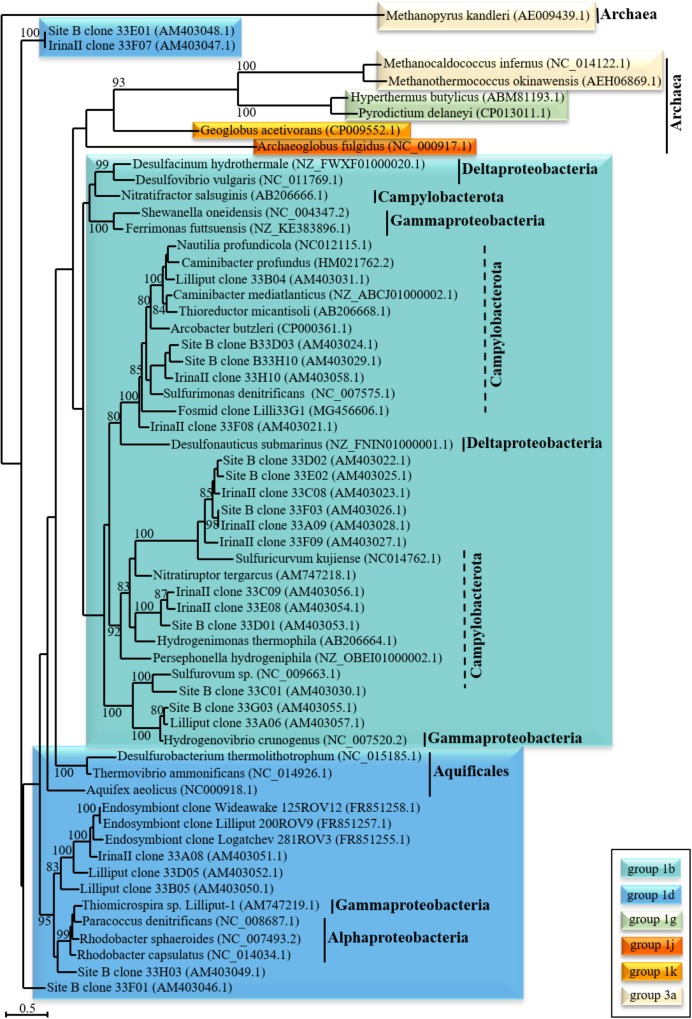
Phylogenetic relationship of “uptake” [NiFe]-hydrogenase large subunit structural genes. The phylogenetic tree was calculated for nucleotide sequences of the large subunit [NiFe]-hydrogenase genes of (primarily vent-derived) phylogenetically diverse Bacteria and Archaea. The scale bar denotes the number of substitutions per nucleotide position and bootstrap values are only indicated if greater than 80%. ClustalW alignments were performed prior to tree calculation using BioEdit (Hall, 1999) with the standard settings. The tree was calculated using seaview (Gouy et al., 2010) with maximum likelihood analysis (four rate classes) and bootstrap values were calculated with 100 replicates. The classification of the [NiFe]-hydrogenase genes was determined using the HydDB web tool (Sondergaard et al., 2016).

Since covering all aspects of microbial hydrogen conversion at hydrothermal vents in detail would go beyond the scope of this review, we will here primarily focus on autotrophic hydrogen-oxidizers. Genes encoding hydrogen-oxidizing (or producing) enzymes have been identified via (meta-)genomic approaches in *Alpha*-, *Beta*-, *Gamma*-, and *Deltaproteobacteria*, *Epsilonproteobacteria* (in the following referred to as Campylobacterota as recently proposed by Waite, 2018), *Firmicutes*, *Actinobacteria*, *Bacteroidetes*, *Aquificales* and other, (less abundant) bacterial and also archaeal phyla in diverse habitats (cf. Figure 2 and Greening et al., 2016). Consistent with the generally great abundance of *Campylobacterota* at hydrothermal vents (often constituting more than 90% of the microbial vent communities in incubation experiments or metagenomic studies) (e.g., Dahle et al., 2013; Perner et al., 2013a; McNichol et al., 2018), a large part of the hydrothermal vent-derived hydrogen oxidizing, autotrophic isolates are related to this class. They are characterized by versatile metabolisms and only a few isolates are strict hydrogen oxidizers (i.e., they are not capable of using any other tested organic or inorganic electron donor), such as the mesophilic *Sulfurovum aggregans* (Mino et al., 2014) or the thermophilic *Caminibacter hydrogeniphilus* (Alain et al., 2002). Overall, there is a trend in the use of alternative electron donors with respect to the thermal preferences: while thermophilic members of the order *Nautiliales* tend to use formate (e.g., Nagata et al., 2017), mesophilic *Campylobacterota* like *Sulfurimonas paralvinellae* have the ability to use different reduced sulfur species such as thiosulfate or elemental sulfur as energy sources (Takai et al., 2006). Based on their metabolic and physiological versatility, *Campylobacterota* occupy diverse niches and can dominate microbial communities in hydrothermal vent environments. The frequent isolation of H_2_-oxidizing *Campylobacterota* from deep-sea vents further emphasizes that this class may play a major role in hydrogen conversion and hydrogen-based primary production within hydrothermal habitats (Corre et al., 2001; Nakagawa et al., 2005; Campbell et al., 2006).

Hydrogen-oxidizing *Deltaproteobacteria* isolated from deep-sea vents – like *Desulfonauticus submarinus* – are commonly heterotrophic (Audiffrin et al., 2003), albeit representatives of this class were isolated from deep-sea vents that can couple hydrogen oxidation to autotrophic growth. Up to now, the vent-derived autotrophic, hydrogen-oxidizing *Deltaproteobacteria* are nearly all characterized as thermophiles with temperature optima between 50 and 61°C, with the so far only exception being a *Desulfobulbus* species with a mesophilic temperature optimum (Sievert and Kuever, 2000; Slobodkin et al., 2013; Slobodkina et al., 2016; Han et al., unpublished). Notably, among them the thermophilic *Desulfacinum hydrothermale* belongs to the group of Sulfate Reducing Bacteria (SRB). Most members of the SRB (which are ubiquitously found in anoxic habitats) belong to the *Deltaproteobacteria* and the group of SRB is known for comprising autotrophs that couple the oxidation of hydrogen to the reduction of sulfate or other electron acceptors as thiosulfate or elemental sulfur (Sievert and Kuever, 2000; Muyzer and Stams, 2008). As the substrates for hydrogenotrophic growth of SRB are readily available in hydrothermal vent systems, deltaproteobacterial SRB may contribute to hydrogen consumption in anoxic hydrothermal vent habitats to a greater extent than previously thought. Still, this hypothesis needs to be proven by the continuing identification of such microorganisms. So far, further evidence for the presence of hydrogen-converting *Deltaproteobacteria* in hydrothermal fluids stems from metatranscriptomic data, where deltaproteobacterial genes encoding hydrogen-converting enzymes were identified (Fortunato and Huber, 2016). *Gammaproteobacteria* are also demonstrated to be relevant for microbially mediated hydrogen cycling. The gammaproteobacterial *Thiomicrospira/Hydrogenovibrio*/*Thiomicrorhabdus* genera (recently reclassified by Boden et al., 2017) can be significantly enriched in bacterial vent communities with relative abundances of up to 37% based on 16S rRNA sequencing (Brazelton and Baross, 2010; Brazelton et al., 2010; Perner et al., 2011a). For many years isolates of the *Thiomicrospira* group (some of which are regrouped into the *Hydrogenovibrio* genus) were described as conventional sulfur oxidizers (Brinkhoff et al., 1999; Takai et al., 2004a; Knittel et al., 2005), until the first sequenced genome of this group indicated genes encoding hydrogen-converting enzymes (Scott et al., 2006) and strains of this group were shown to use hydrogen (Hansen and Perner, 2015, 2016). Although other hydrogen-oxidizing, autotrophic, gammaproteobacterial isolates have not been recovered yet, there is evidence for the hydrogen-converting ability among this group, based on classical sulfur-oxidizing symbionts (Petersen et al., 2011) and widespread deep-sea bacteria of the SUP 05 clade (Anantharaman et al., 2016).

Besides members of the Proteobacteria, other Bacteria and also Archaea contribute to the hydrogen-oxidizing communities in deep-sea vents. Particularly (among the Bacteria) the deeply branching order of *Aquificales* hosts a wide range of hydrogen-oxidizing organisms of different families and genera (e.g., *Desulfurobacteriaceae*) that have been isolated from hydrothermal fields around the globe (L’Haridon et al., 2006; Ferrera et al., 2014). Although they differ in their specific growth requirements (e.g., temperature, pH, electron acceptors), they are all described as strict chemolithoautotrophs and thermophiles (Nakagawa et al., 2003; L’Haridon et al., 2006). The strictly anaerobic, vent-derived *Desulfurobacterium thermolithotrophum* from the family of the *Desulfobacteraceae* was the first known thermophilic bacterial isolate with the ability to act as a primary producer in the temperature range of 45–70°C (L’Haridon et al., 1998). Notably, most members of the *Aquificales*, such as *Thermovibrio ammonificans* or *Balnearium lithotrophicum*, use hydrogen as the only energy source for autotrophic growth (Takai et al., 2003; Vetriani et al., 2004). Another thermophile-comprising phylum, *Thermodesulfobacteria*, usually is not considered as an important contributor of hydrogen-oxidizing vent-derived bacteria. Nonetheless, two of its five genera comprise thermophilic SRB isolated from hydrothermal vents, which share the ability to use hydrogen as the sole energy source for autotrophic growth (Jeanthon et al., 2002; Moussard et al., 2004; Alain et al., 2010).

Among the Archaea, thermophilic and hyperthermophilic methanogens are supposed to be the numerically largest and (in terms of the hydrogen consumption ability) most important group of hydrogen-oxidizers in hotter temperature regimes (Huber et al., 2002; Topcuoglu et al., 2016; Fortunato et al., 2018). Starting from the 1980’s, shortly after the first discovery of hydrothermal vent systems, methanogenic isolates were repeatedly drawn from hydrothermal vents (e.g., Huber et al., 1982, 1989; Jones et al., 1989), some of them using hydrogen as the sole energy source for autotrophic CO_2_ fixation and methane production (e.g., Huber et al., 1982; Jeanthon et al., 1998; L’Haridon et al., 2003; Takai et al., 2004b). As mentioned above, the hydrogen can stem from an abiogenic source (e.g., resulting from serpentinization processes) or be produced by hydrogen-evolving microorganisms (Figure 1, Ver Eecke et al., 2012; Toki et al., 2016; Topcuoglu et al., 2016). Methanogenic communities require greater hydrogen concentrations (e.g., ≥17 μM according to experiments with hyperthermophilic *Methanocaldococcus* species) to support chemolithoautotrophic growth than organisms coupling hydrogen oxidation to alternative electron acceptors, e.g., oxygen, nitrate, ferric iron and sulfate (Lovley and Goodwin, 1988; Hoehler et al., 1998; Ver Eecke et al., 2012). Besides methanogens, other hydrogen-oxidizing, autotrophs also exist among the Archaea: Fe(III)-reducing, hydrogen-oxidizing hyperthermophiles are encountered among the *Euryarchaeota* and *Crenarchaeota* (Slobodkina et al., 2009; Lin et al., 2016). These hydrogenotrophic Archaea thrive at lesser hydrogen concentrations than methanogens (Ver Eecke et al., 2009). Thus, they may be important contributors to microbial hydrogen consumption in venting environments that are characterized by elevated temperatures but lesser hydrogen levels. Nonetheless, the biogeochemical and ecological impact of these two groups still needs to be resolved.

Despite the large difficulties typically associated with taking samples from deep-sea hydrothermal vents and the culturing of vent-derived microorganisms, a large number of hydrogen-oxidizers has been isolated so far. However, a decreasing trend can be observed regarding the number of novel isolates from hydrothermal vent environments, which may be caused by insuperable obstacles in defining the appropriate culture conditions. More likely though, the laborious efforts in isolating (extremophilic) slow-growing microorganisms from hydrothermal vents have lessened due to the advent of cost-effective culture-independent techniques. For now, we have only gained a small-scale insight into the great diversity of microbial hydrogen uptake taking place at hydrothermal vents (see further discussions below).

## Hydrogenase Genes

The interconversion of molecular hydrogen to protons and electrons (H_2_ ↔ 2H^+^ + 2e^-^) is catalyzed by hydrogenase enzymes, which are widely distributed among Bacteria and Archaea. Hydrogenases are classified according to their catalytic center and to date three different types are known: (i) [NiFe]-hydrogenases, (ii) [FeFe]-hydrogenases and (iii) [Fe]-hydrogenases (Vignais and Billoud, 2007). [NiFe]-hydrogenases are usually involved in hydrogen sensing and consumption, [FeFe]-hydrogenases are the so-called “hydrogen-evolving” (producing) hydrogenases and [Fe]-hydrogenases play a key role in methanogenesis (Thauer, 1998; Vignais and Billoud, 2007). Among the [NiFe]-hydrogenases four groups are distinguished, that each can be further divided into several subgroups based on different parameters concerning the catalytic subunit like amino acid sequence phylogeny and reported biochemical properties. Group 1 and group 4 [NiFe]-hydrogenases are termed membrane-bound “H_2_-uptake” (consuming) and “hydrogen-evolving” hydrogenases, respectively, which are involved in energy metabolism. The group 2 encompasses mainly cytosolic hydrogen-sensing hydrogenases and some with so far unknown function and localization, while the cytosolic group 3 includes the F_420_-reducing hydrogenases from methanogens, the bifunctional NADP-coupled hydrogenases and the bifurcating, heterodisulphide-linked hydrogenases (Greening et al., 2016; Sondergaard et al., 2016).

The [FeFe]-hydrogenases can also be further distinguished in three groups (A-C), of which groups A and C are additionally subdivided into four and three subgroups, respectively. Notably, only group A1 hosts the prototypical “hydrogen-evolving” [FeFe]-hydrogenases (other group A hydrogenases are involved in electron bifurcation or have unknown functions). [FeFe]-hydrogenases of groups B and C are currently only assigned to putative functions involved in hydrogen sensing and hydrogen production (Sondergaard et al., 2016).

While [NiFe]- and [FeFe]-hydrogenases are present in diverse prokaryotes, [Fe]-hydrogenases are only found in methanogenic Archaea and cannot be subdivided into distinct groups (Greening et al., 2016; Sondergaard et al., 2016). In contrast to [NiFe]- and [FeFe]-hydrogensaes, they do not contain FeS-clusters and couple the oxidation of hydrogen to the reduction of methenyltetrahydromethanopterin. This intermediary step is only required in the reduction of CO_2_ to methane under nickel limiting conditions when [NiFe]-hydrogenases cannot be synthesized (Vignais and Billoud, 2007). Due to the habitat-specific conditions, “uptake” [NiFe]-hydrogenases (and primarily those of the prototypical group 1b, see Figure 2) likely are the most common and (concerning primary biomass production) important hydrogenase type in deep-sea hydrothermal vent systems. As hydrothermal fluids usually contain various minerals and metals, nickel (central component of the active center in [NiFe]-hydrogenases) limitation should not occur in these habitats. Moreover, given the elevated hydrogen concentrations (>μM levels), fermentative hydrogen production is likely limited or inhibited in hydrothermal vent systems, leading to the assumption that microbial hydrogen oxidation (catalyzed by [NiFe]-hydrogenases) may be the dominating process in these environments.

**FIGURE 3:**
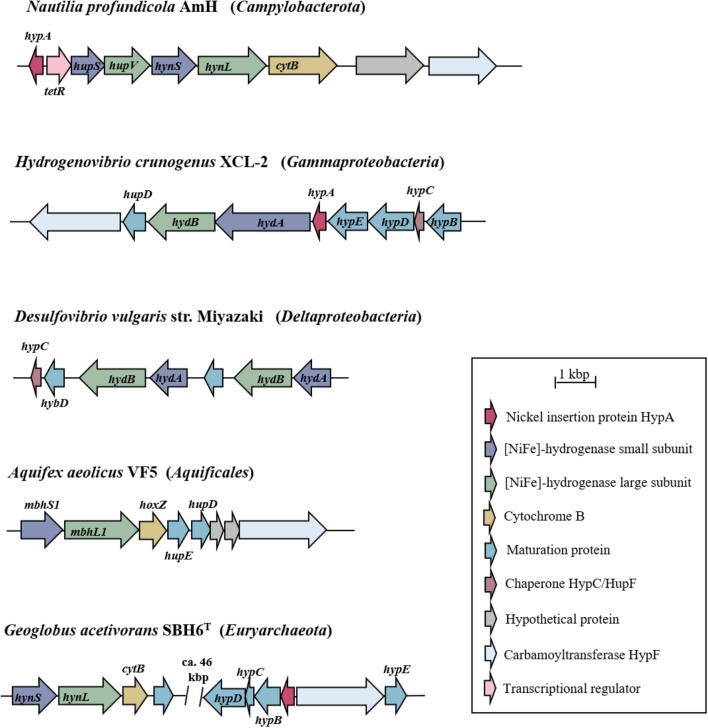
Hydrogenase gene clusters of bacterial and archaeal representatives. Only the gene clusters containing the structural genes for the large and small subunit of the [NiFe]-hydrogenases and the corresponding maturation proteins are shown. According to the classification of Sondergaard et al. (2016) the [NiFe]-hydrogenases of *N. profundicola*, *H. crunogenus*, and *D. vulgaris* belong to group 1b, that of *A. aeolicus* to group 1d and that of *G. acetivorans* to group 1k (cf. Figure 2). Genes are pictured as arrows in the direction of transcription. Arrows of the same color indicate the same function of the encoded protein as explained by the key legend. Gene (and protein) abbreviations follow the respective annotations in the publicly available databases.

Hydrogenase genes (and those of [NiFe]-hydrogenases in particular) are usually arranged in gene clusters that differ in their size and gene patterns (Figure 3). Due to the highly specific and complex maturation processes involved in the biosynthesis of hydrogenases, the clusters (in addition to the catalytic subunits) commonly also comprise genes encoding proteins for electron transfer, regulation factors and maturation factors, but also hypothetical proteins and partner enzymes (Casalot and Rousset, 2001; Bock et al., 2006; Greening et al., 2016). Commonly, the heterologous expression of (hydrogenase) enzymes (i.e., the expression in a foreign host) is limited by promoter recognition, diverging codon-usage, translation and the incompatibility or a lack of the respective maturation and assembly apparatus (cf. Perner et al., 2011b). In *E. coli* for example, the exchange of a carboxy-terminal extension of the large subunit of a [NiFe]-hydrogenase with that from an isoenzyme resulted in the abortion of the protein maturation. This indicates the great specificity of the proteolytic cleavage by the endopeptidase HybD, which is a necessity to form an active hydrogenase (Theodoratou et al., 2000; Casalot and Rousset, 2001). Furthermore, nickel incorporation proteins (HypA) or the carbamoyltransferase HypF (involved in the formation of the active site) are of vital importance for the formation of a functional protein (Figure 3, Casalot and Rousset, 2001). Nevertheless, heterologous expression of [NiFe]-hydrogenases has successfully been demonstrated in the past: not only with genes of (phylogenetically) closely related organisms (Rousset et al., 1998) but also in a setup where the insert hydrogenase and the host stem from different bacterial classes (Adam and Perner, 2018).

Hydrogenase genes from hydrothermal vents have been targeted by PCR amplification (group 1 and F_420_-reducing [NiFe]-hydrogenases) (Takai et al., 2005; Perner et al., 2010; Petersen et al., 2011) or by direct sequencing of metagenomes (Perner et al., 2014; Pjevac et al., 2018) and metatranscriptomes (Dahle et al., 2013; Fortunato and Huber, 2016) (consisting of the whole genetic information) of vent-derived samples. However, compared to some enzymes like esterases, which are considered as one of the most important industrial biocatalysts (cf. Perner et al., 2011b), hydrogenases have only rarely been in the focus of metagenomic studies. Moreover, in most cases the metagenomic datasets were merely screened for the presence of [NiFe]-hydrogenase genes (e.g., Brazelton et al., 2012; Dahle et al., 2013; Perner et al., 2014; Fortunato and Huber, 2016). The majority of the identifiable [NiFe]-hydrogenase genes of these studies can be phylogenetically related to members of the *Campylobacterota* (see phylogenetic tree, Figure 2), other proteobacterial and also archaeal phyla. The frequent identification of *Campylobacterota* may be a consequence of the specific primer sets and the database entries that are available to identify hydrogenase genes. As only those genes can be identified that share sequence homologies to known hydrogenase sequences (or specific groups), it cannot be excluded that by applying PCR- and other sequence-based techniques an unintentional selection occurred. Still, the large number of campylobacterotal isolates also emphasizes the importance for primary biomass production and large abundance of this group in hydrothermal vent systems.

However, examples exist where no campylobacterotal genes could be identified: the [NiFe]-hydrogenase genes identified in the metagenome of a chimney sample from the hydrogen-rich, ultramafic Lost City hydrothermal field were primarily affiliated with betaproteobacterial [NiFe]-hydrogenase genes, showing the greatest resemblance to the *Ralstonia eutropha* hydrogenase (Brazelton et al., 2012). Since *R. eutropha* and other *Betaproteobacteria* closely related to the hydrogenases found in the Lost City metagenome are aerobic or facultatively anaerobic, the authors assume that the respective hydrogen oxidizers thrive in the oxic-anoxic transition zone of the chimney. Surprisingly, in addition to these [NiFe]-hydrogenases, “hydrogen-evolving” [FeFe]-hydrogenases related to *Clostridiales* were identified, which are most likely associated with fermentation of organic compounds. Given that fermentative hydrogen production can be inhibited even at nM hydrogen concentrations (discussed earlier), microbial hydrogen production technically does not seem feasible in a hydrogen-rich vent environment like the Lost City hydrothermal field. Furthermore, the origin of the organic fermentation substrates has not been resolved yet. Still, there are indications that in the course of serpentinization processes abiogenic organic carbon might evolve that can be used in microbial fermentation. As most fermentation processes require anoxic conditions, it is proposed that anaerobic *Clostridia* colonize the anoxic, deep-subsurface of the Lost City vent system (eventually entrained to the surface as the fluids pass by). It may also be possible that the hydrogen produced by *Clostridia* is later consumed by the hydrogen oxidizing *Betaproteobacteria* (Brazelton et al., 2012 and references therein). Despite the metagenomic indications, clear evidence for hydrogen pathways (including the oxidation of microbially produced hydrogen) in this habitat is still missing (cf. Figure 1). So far, it remains unclear if the respective genes actually belong to living organisms and are transcribed and expressed as functioning enzymes. Recently, Pjevac et al. (2018) stated a large discrepancy in the hydrogenase frequency of metagenomes and metaproteomes of two distinct chimneys of the Roman Ruins vent field: From 160 phylogenetically diverse hydrogenase genes identified in the metagenomes only five proteins were found in the respective metaproteomes, all belonging to campylobacterotal representatives. Accordingly, the great phylogenetic diversity (in general but also of the hydrogenase genes) does not coincide with the actual metabolic diversity of the microbial communities. This phenomenon may in part be explained by the fact that due to the great microbial diversity, protein quantities of hydrogenases probably lay below the detection threshold of the experimental setup. Additionally, many of the “uptake” hydrogenases are membrane-associated and a bias of the isolation method against such membrane-bound proteins has to be considered. Still, it cannot be excluded that a significant part of the hydrogenase genes present in the metagenome are not expressed and are therefore missing in the metaproteome (Pjevac et al., 2018).

A deepened insight into putatively active metabolic processes – and microbial hydrogen utilization – as well as possible regulating factors can also be gained by metaproteomic approaches (Anantharaman et al., 2013; Fortunato et al., 2018). A comparison of the metatranscriptomes of a plume and a background sample of the Guaymas basin demonstrated site-specific (up-regulated) transcript abundances of distinct [NiFe]-hydrogenase genes of plume-derived and epipelagic members of the sulfur-oxidizing SUP05 group of the *Gammaproteobacteria*. Combined with transcript abundances of other genes related to chemoautotrophy like a form II ribulose-1,5-bisphosphate carboxylase-oxygenase (RubisCO) and other genes of the Calvin-Benson-Bassham (CBB) cycle, the hydrogenase expression levels indicate that hydrogen oxidation strongly contributes to the energy budget of the SUP05 group thriving in deep-ocean habitats (Anantharaman et al., 2013). Gammaproteobacterial [NiFe]-hydrogenase genes and transcripts were – in addition to those of *Deltaproteobacteria* and *Campylobacterota* as well as the methanogenic F_420_-reducing hydrogenase - also detected in metagenomes and metatranscriptomes of low-temperature diffuse fluid samples derived from the Axial Seamount hydrothermal field (Fortunato and Huber, 2016). In this study meta-omics were coupled to additional, hydrogen-enriched, RNA stable isotope probing incubation experiments at different temperatures, integrating the influence of a thermal gradient on the chemoautotrophic microbial community. This thermal gradient was reflected by the hydrogen-oxidizing communities of the incubation experiments: while at 30°C exclusively (mesophilic) *Campylobacterota* were found, at 55°C thermophilic *Campylobacterota* and (to a lesser extent) methanogens dominated. The 80°C incubations, however, were dominated by hyperthermophilic methanogens, indicating that methanogenesis was the main metabolism at this temperature and may play a significant role for primary production in subsurface habitats, characterized by greater temperatures and greater hydrogen concentrations (Fortunato and Huber, 2016). In a more recent metaproteomic study, Fortunato and co-workers compared the fluid communities of three hydrothermal vents of the Axial Seamount field, sampled on an annual basis over a period of 3 years. Fluids from Marker 33 and Marker 113 exhibited 10 to 30 times lesser hydrogen concentrations than that of the Anemone vent. However, the hydrogen-poor fluids exhibited greater abundances and expression levels of hydrogenase genes and a greater percentage of hydrogen-utilizing *Campylobacterota*, *Aquificae* and methanogens. Notably, methanogenic transcripts at Marker 113 ranged from 30 to 56% of all annotated transcripts, with a large portion of hydrogenase genes. Therefore, the lesser hydrogen concentrations are likely caused by a draw-down of hydrogen through microbial hydrogen oxidation. Overall, more than 90% of the intra- and inter-vent changes in the community compositions observed within this study could be explained with the geochemical variables determined for the different fluid, plume and background samples (e.g., temperature, pH, hydrogen, sulfide and nitrate concentrations) (Fortunato et al., 2018).

Yet, such clear-cut, proportional relations between abiotic environmental parameters and the corresponding microbial (metabolic) diversity are often difficult to draw. In particular, differing hydrogen-concentrations are often not directly reflected by the microbial community: varying hydrogen concentrations, for example, do not necessarily lead to differences in the diversity and abundance of hydrogenase genes. The hydrogenase distribution across differing hydrogen concentrations indicates that other environmental parameters also play a central role in the distribution of hydrogen oxidizing microorganisms (Perner et al., 2010, 2014). Other factors putatively influencing the diversity and abundance of hydrogenase genes observed in hydrothermal vent environments might be the kinetics and affinities of the respective enzymes. The K*_m_* values of [NiFe]-hydrogenases reported in the past show a great diversity ranging from 0.06 to 140 μM (Léger et al., 2004; van Haaster et al., 2005 and references therein). It may be assumed that organisms harboring high-affinity hydrogenases exhibiting low K*_m_* values can suppress hydrogen oxidizers that harbor hydrogenases with greater K*_m_* values, leading to a reduced diversity. However, the high-affinity, oxygen-tolerant [NiFe]-hydrogenases of group 1 h/5, which are widely distributed in soils (Constant et al., 2011), have not been identified in hydrothermal vent environments yet.

Furthermore, a metatranscriptomic study showed that increased hydrogenase gene expression is not limited to hydrothermal emission zones with elevated hydrogen concentrations but can also be observed at similar levels in intra-field water samples. The latter are not directly hydrothermally influenced but located in the vicinity of diffuse venting sites (Olins et al., 2017). Compared to background water samples, in most diffuse fluids and intra-field water samples the hydrogenase transcript levels were significantly enriched (Olins et al., 2017). The frequent identification of hydrogenase genes and elevated hydrogenase transcript abundances in hydrothermal vents and intra-field waters give evidence that hydrogen oxidation is of particular importance for primary biomass production in the different habitats surrounding hydrothermal vent orifices.

## Hydrogen Consumption Measurements

Despite influences of individual fluid composition and seawater mixing ratios, compared to hydrogen-poor basalt-hosted systems, microbial hydrogen consumption rates of hydrogen-rich, ultramafic-hosted vent systems generally are expected to be greater. In fact, *ex situ* incubation experiments with symbiont-hosting mussel tissue from distinct vent systems revealed a 20- to 30-fold greater hydrogen consumption potential of symbionts from the hydrogen-rich ultramafic vent system relative to the hydrogen-poor basalt-hosted system (Table 2, Petersen et al., 2011). The respective CO_2_-fixation rates confirmed that hydrogen oxidation fueled autotrophy (Table 2, Petersen et al., 2011). *Ex situ* incubations with diverse hydrothermal fluids (and free-living microorganisms), however, could not confirm the thermodynamic estimates. In most incubations, hydrogen consumption rates and biomass production were greater in the tested fluids from basaltic than from ultramafic systems. These observations may result from the specific conditions provided with the experimental setup, i.e., oxic and anoxic conditions, addition of 12–14 μM hydrogen (in solution) and incubation at 18°C (Perner et al., 2010, 2011a, 2013b). Accordingly, altered incubation conditions may exhibit quite different hydrogen consumption rates. Similar incubation experiments, performed with only basalt-hosted hydrothermal emissions, were advanced by mimicking *in situ* pressure and temperature in gas-tight samplers (McNichol et al., 2016). Nitrate availability had a stimulating effect on the respective hydrogen consumption rates, ranging from 3.66 to 63.97 fmol H_2_ cell^-1^ h^-1^ (Table 2, McNichol et al., 2016), comparable to those of previous *ex situ* measurements ranging from 0.2 to 92.0 fmol H_2_ cell^-1^ h^-1^ (Perner et al., 2013b). Despite the efforts made to reproduce *in situ* conditions in *ex situ* incubations, it is impossible to simulate the dynamic nature of the (micro) habitats present in the hydrothermal vent systems. These are hallmarked by vast thermal and chemical gradients in venting habitats, ranging from several 100 s to 4°C water temperature and from highly reduced to fully oxic, respectively. Therefore, incubations with more conditions than manageable would have to be set up to cover all the micro niches present in a hydrothermal venting biotope (Perner et al., 2010). Other methods to determine the microbial hydrogen oxidation potential, e.g., the tritium-based hydrogenase assay applied to subsurface sediments also show a great potential for hydrogen oxidation (Adhikari et al., 2016). Yet, they suffer from similar limitations as the hydrogen consumption measurements of hydrothermal vent samples: the incubation experiments are not conducted under *in situ* conditions and freezing of the samples prior to the assay cause additional deviation (Adhikari et al., 2016). Against this background, the development of *in situ* techniques for the determination of microbial hydrogen consumption rates is inevitable.

**Table 2:** Hydrogen consumption rates of different *ex situ* measurements performed with hydrothermal fluid samples or bacterial strains isolated from hydrothermal environments.

Sample type or strain	T	O_2_	H_2_ addition	Other incubation characteristics	H_2_ consumption rate	CO_2_-fixation rate	Reference
Wideawake diffuse fluids, basalt-hosted, MAR	18°C	+	+ 2% in head space		13.9 ± 1.7 – 18.9 ± 3.1 [fmol H_2_ cell^-1^ h^-1^]	0.1 – 0.2 [fmol CO_2_ cell^-1^ h^-1^]	Perner et al., 2013a
		–			63.7 ± 24.0 – 89.0 ± 25.9 [fmol H_2_ cell^-1^ h^-1^]	0.1 – 0.2 [fmol CO_2_ cell^-1^ h^-1^]	
Clueless diffuse fluids, basalt-hosted, MAR	18°C	+	+ 2% in head space		0.1 ± 0.08 [fmol H_2_ cell^-1^ h^-1^]	0.004 [fmol CO_2_ cell^-1^ h^-1^]	
		–			0.01 ± 0.004 [fmol H_2_ cell^-1^ h^-1^]	<0.0001 [fmol CO_2_ cell^-1^ h^-1^]	
Desperate diffuse fluids, basalt-hosted, MAR	18°C	+	+ 2% in head space		0.2 ± 0.1 [fmol H_2_ cell^-1^ h^-1^]	0.0002 [fmol CO_2_ cell^-1^ h^-1^]	
		–			0.09 ± 0.02 [fmol H_2_ cell^-1^ h^-1^]	0.0005 [fmol CO_2_ cell^-1^ h^-1^]	
Sisters Peak diffuse fluids, basalt-hosted, MAR	18°C	+	+ 2% in head space		0.8 ± 0.05 [fmol H_2_ cell^-1^ h^-1^]	<0.001 [fmol CO_2_ cell^-1^ h^-1^]	
		–			49.3 ± 6.1 [fmol H_2_ cell^-1^ h^-1^]	0.2 ± 0.1 [fmol CO_2_ cell^-1^ h^-1^]	
Foggy Corner diffuse fluids, basalt-hosted, MAR	18°C	+	+ 2% in head space		82.0 ± 10.0 [fmol H_2_ cell^-1^ h^-1^]	0.003 ± 0.001 [fmol CO_2_ cell^-1^ h^-1^]	
		–			92.0 ± 11.0 [fmol H_2_ cell^-1^ h^-1^]	0.006 ± 0.001 [fmol CO_2_ cell^-1^ h^-1^]	
Lilliput diffuse fluids, basalt-hosted, MAR	18°C	+	+ 2% in head space		0.3 ± 0.06 [fmol H_2_ cell^-1^ h^-1^]	0.01 [fmol CO_2_ cell^-1^ h^-1^]	
		–			0.3 ± 0.004 [fmol H_2_ cell^-1^ h^-1^]	0.01 [fmol CO_2_ cell^-1^ h^-1^]	
Quest diffuse fluids, ultramafic-hosted, MAR	18°C	+	+ 2% in head space		<0.002 [fmol H_2_ cell^-1^ h^-1^]	0.002 ± 0.001 [fmol CO_2_ cell^-1^ h^-1^]	
		–			<0.02 [fmol H_2_ cell^-1^ h^-1^]	0.002 ± 0.001 [fmol CO_2_ cell^-1^ h^-1^]	
Irina II diffuse fluids, ultramafic-hosted, MAR	18°C	+	+ 2% in head space		17.0 ± 17.1 [fmol H_2_ cell^-1^ h^-1^]	0.02 [fmol CO_2_ cell^-1^ h^-1^]	
		–			1.6 ± 1.9 [fmol H_2_ cell^-1^ h^-1^]	0.02 [fmol CO_2_ cell^-1^ h^-1^]	
Irina II plume, ultramafic-hosted, MAR	18°C	+	+ 2% in head space		50.5 ± 14.6 [fmol H_2_ cell^-1^ h^-1^]	0.001 [fmol CO_2_ cell^-1^ h^-1^]	
		–			2.6 ± 2.0 [fmol H_2_ cell^-1^ h^-1^]	0.001 [fmol CO_2_ cell^-1^ h^-1^]	
Nibelungen hot fluids, ultramafic-hosted, MAR	18°C	+	+ 2% in head space		0.2 ± 0.1 [fmol H_2_ cell^-1^ h^-1^]	0.003 [fmol CO_2_ cell^-1^ h^-1^]	
		–			0.7 ± 0.04 [fmol H_2_ cell^-1^ h^-1^]	0.003 [fmol CO_2_ cell^-1^ h^-1^]	
Crab Spa diffuse fluids, basalt-hosted, EPR	24°C	Not added	150 μM dissolved H_2_	25 MPa pressure	3.66 – 5.77 [fmol H_2_ cell^-1^ h^-1^]	n.d.	McNichol et al., 2016
Crab Spa diffuse fluids, basalt-hosted, EPR	24°C	Not added	150 μM dissolved H_2_	25 MPa pressure + 100 μM nitrate	14.65 – 21.18 [fmol H_2_ cell-1 h^-1^]	n.d.	
Crab Spa diffuse fluids, basalt-hosted, EPR	50°C	Not added	150 μM dissolved H_2_	25 MPa pressure + 100 μM nitrate	41.24 – 63.97 [fmol H_2_ cell^-1^ h^-1^]	n.d.	
Symbiont-hosting *Bathimodiolus* tissue from ultramafic Logatchev field, MAR	4°C	+	100 ppm in head space		656 ± 207 [nmol H_2_ h^-1^ (g wet weight)^-1^]	∼ 67 [^14^C Bq (g wet weight)^-1^]	Petersen et al., 2011
Symbiont-hosting *Bathimodiolus* tissue from ultramafic Logatchev field, MAR	4°C	+	100–1783 ppm in head space		656 ± 207 – 2945 ± 201 [nmol H_2_ h^-1^ (g wet weight)^-1^]	n.d.	
Symbiont-hosting *Bathimodiolus* tissue from basalt-hosted Comfortless Cove field, MAR	4°C	+	95–938 ppm in head space		30 ± 25 – 208 ± 67 [nmol H_2_ h^-1^ (g wet weight)^-1^]	n.d.	
Symbiont-hosting *Bathimodiolus* tissue from basalt-hosted Lilliput field, MAR	4°C	+	93–2916 ppm in head space		20 ± 9 – 316 ± 100 [nmol H_2_ h^-1^ (g wet weight)^-1^]	n.d.	
Janssand sediments, German Wadden Sea	14°C	–	220 μM in head space		0.46 [fmol H_2_ cell^-1^ h^-1^]	n.d.	Dyksma et al., 2018
*Hydrogenovibrio* SP-41^∗^	28°C	+	2% in head space	Different growth media were tested	1.47 – 6.1 [fmol H_2_ cell^-1^ h^-1^]	n.d.	Hansen and Perner, 2015
*H. crunogenus* TH-55^∗^	28°C	+	2% in head space		0.73 [fmol H_2_ cell^-1^ h^-1^]	n.d.	


*T states the incubation temperature during the experiments and O_2_ indicates whether the experiments were conducted under oxic (+) or anoxic (-) conditions. For comparison, a non-hydrothermal sample (Janssand sediments) is also included. ^∗^Previously *Thiomicrospira* (*crunogena*).*

*In situ* measurements of hydrogen concentrations are already being done by employing *in situ* mass spectrometry (Wankel et al., 2011; Perner et al., 2013a) and has been used to draw conclusions on the impact of subsurface microbial activity on hydrogen concentrations of diffuse hydrothermal fluids. A discrepancy between the calculated and actually measured hydrogen concentrations of hydrothermal fluids, ranging from 50 to 80%, was attributed to microbial activity taking place below the seafloor (Wankel et al., 2011). Yet, a link to the microorganisms responsible for the presumable hydrogen consumption is missing. To provide this link, the existing measurement techniques could be amended by the recently established *in situ* fixation of fluids for later nucleotide extraction and metatranscriptomic and/or metagenomic analysis (Fortunato and Huber, 2016; Olins et al., 2017). Therefore, future *in situ* hydrogen measurements and consumption experiments would ideally combine monitoring of hydrogen and CO_2_ concentrations, cell counting and fixation of (fluid) samples for metagenomic and metatranscriptomic analysis to cover the full hydrogen consumption potential of vent-associated microbial communities.

So far, *ex situ* hydrogen consumption measurements have been linked to unspecified *Campylobacterota* (McNichol et al., 2016), mesophilic *Alpha-, Beta*- and *Gammaproteobacteria*, mesophilic *Campylobacterota*, methanogens (Perner et al., 2010, 2011a) as well as a typically sulfur-oxidizing gammaproteobacterial symbiont (Petersen et al., 2011). First hints that another vent-inhabiting, sulfur-oxidizing *Gammaproteobacterium* might be able to oxidize hydrogen were gained from sequencing the hydrogenase gene cluster containing genome of *Thiomicrospira crunogena* (Scott et al., 2006) (now *Hydrogenovibrio crunogenus*) (Boden et al., 2017). Additionally, in some oxic, H_2_-amended incubation experiments, genes related to the sulfur-oxidizing gammaproteobacterial *Hydrogenovibrio crunogenus* were highly (8 to 23-fold) enriched compared to sulfide-spiked incubations of the same vent sample (Perner et al., 2011a). Still, it remained unclear whether *Thiomicrospira* strains actually express functional hydrogenases until *Thiomicrospira* SP-41’s hydrogen consumption ability was discovered (Hansen and Perner, 2015). Further hydrogen consuming (previously classified as) *Thiomicrospira* strains were detected after offering diverse growth conditions and supplements (Hansen and Perner, 2016), demonstrating an unexpected potential for hydrogen consumption among these sulfur-oxidizing *Gammaproteobacteria*. The flexibility to use hydrogen as an alternative electron donor might also be a key to the success and dominance of (other) sulfur-oxidizing *Gammaproteobacteria* as observed in a variety of hydrothermal fluids (Perner et al., 2010; Olins et al., 2017).

A similar hydrogen consumption potential can also be expected for sulfur-oxidizing representatives of the order *Campylobacterales*: for example, the growth of a sulfur-oxidizing *Sulfurimonas denitrificans* isolate was significantly improved by the addition of hydrogen in growth experiments and hydrogen consumption measurements confirmed the utilization as electron donor (Han and Perner, 2014). Although *S. denitrificans* was originally isolated from Wadden Sea sediments (Timmer-Ten Hoor, 1975), numerous strains have also been identified in hydrothermally influenced habitats (cf e.g., Perner et al., 2013a). Furthermore, hydrogenase genes of a *S. denitrificans* strains were found in vent-derived metatranscriptomes (Fortunato and Huber, 2016). Given the great abundances of *Sulfurimonas* and other *Campylobacterales* genera like *Arcobacter* in hydrothermal fluids or plumes (Perner et al., 2010, 2013a; Fortunato and Huber, 2016), members of the *Campylobacterales* may contribute significantly to overall hydrogen consumption in deep-sea vent systems.

Although diverse archaeal hydrogen-consuming representatives have been isolated, much of the archaeal hydrogen consumption in hydrothermal vents can likely be assigned to methanogens, evidenced by incubation experiments and sequencing (Perner et al., 2010; Ver Eecke et al., 2012; Fortunato and Huber, 2016). The full potential of methanogenic hydrogen-based primary production, however, may be even greater than current incubation experiments suggest, occurring over a wider temperature and pressure range. By applying elevated (*in situ*) hydrostatic pressure of 20 MPa, the growth range of the hyperthermophilic vent-derived *Methanopyrus kandleri* was expanded from 116°C up to 122°C and the temperature optimum was increased by 5°C to 105°C (compared to the standard 0.4 MPa incubations), while the carbon isotope fractionation of generated methane decreased (Takai et al., 2008). The small carbon isotope fractionation of biogenic methane could lead to a misinterpretation of data from hydrothermal vent environments: it could be identified as isotopically “heavy” methane from a magmatic source, thus diminishing the estimated methanogenic contribution (Takai et al., 2008 and references therein).

So far, incubation experiments with hydrothermal fluid samples have been performed with temperatures up to 80°C (Fortunato and Huber, 2016), thus the conditions might not have been ideal for hyperthermophiles in the existing incubations and their abundances were underestimated.

Before genomic analyses and incubation experiments could link hydrogen consumption to the putatively responsible organisms, for many species such as *Thiomicrospira* sp. no hints for a potential hydrogen utilization were obvious. Matched with the still existing difficulties in the cultivation of vent inhabitants, a need for the implementation of culture-independent approaches becomes evident in order to identify novel hydrogen-oxidizing or -producing microorganisms and respective enzymes.

## Accessing the Uncultured Majority and Their Hydrogen-Converting Potential

Hydrogenase genes have been frequently identified in metagenomic deep-sea hydrothermal vent data sets. The [NiFe]-hydrogenase hit rate (i.e., the number of identified hydrogenase genes relative to all other genes in the data set) from a hydrothermal vent metagenome can be up to 40-fold higher than in metagenomes from other habitat types (Brazelton et al., 2012; Perner et al., 2014), revealing the importance of hydrogen-uptake in venting biotopes. However, these sequence-based approaches only indicate potential hydrogenase encoding genes. The functionality of the putative hydrogen-converting enzymes remains unclear, until hydrogen-uptake or –evolution is experimentally confirmed. Furthermore, hydrogen-converting enzymes lacking sequence homologies to known hydrogenases cannot be identified by sequence-based metagenomic approaches. Up to now, truly novel enzymes can only be found by screening metagenomes with activity-based approaches (Handelsman, 2004).

Until recently no activity-based screen existed, that could seek hydrogen-converting enzymes from the environment. However, a newly developed screen enables the search for environmental hydrogenases: It is based on the recombinant expression of metagenome-derived genes in a [NiFe]-hydrogenase deletion mutant of *Shewanella oneidensis* MR-1 (Adam and Perner, 2017). By applying this screen to metagenomic libraries of hydrothermal vent environments, hydrogen-converting clones were identified, whose metagenomic inserts largely do not share any sequence homology with known hydrogenases. Hydrogen-uptake activities of the clones exhibited up to 258 ± 19 nmol H_2_^∗^min^-1∗^mg^-1^ of partially purified proteins at 55°C, exceeding those of some cultured organisms (Adam and Perner, 2018). Given the difficulties and drawbacks associated with heterologous (hydrogenase) enzyme expression, a limitation in the hydrogenase detection ability of this screen is not surprising. For example, hydrogenases of *Escherichia coli*, *Hydrogenovibrio* sp., *Thiobacillus denitrificans*, *Desulfovibrio vulgaris*, and *Aquifex aeolicus* (and thus likely those of uncultured relatives from the environment) could not be identified with this host-vector system. Nevertheless, the [NiFe]-hydrogenases from *Photobacterium leiognathi*, *Rhodobacter capsulatus*, *Sulfurimonas denitrificans* and *Wolinella succinogenes* were successfully expressed and exhibited measurable activities that were up to 2.6-fold higher than that of the host’s own hydrogenase (Adam and Perner, 2018). Still, it can be assumed that by establishing other hosts with varying growth optima for the activity-based screen, the detection range may be significantly improved. Given specific hydrogen-uptake activities of up to 48,700 ± 4,000 nmol H_2_^∗^min^-1∗^mg^-1^ for the vent isolate *Thioreductor micantisoli* (Takai et al., 2005), the large potential for the discovery of (highly) active hydrogenases present in the enzymatic pool of vent environments is apparent.

The possibility of successfully expressing vent-derived hydrogen-converting enzymes in an “easily” culturable host may also open the door to biotechnological applications of these enzymes. Hydrogen-converting enzymes are of particular interest for the use in hydrogen production as a clean energy carrier and energy generation in biofuel cells (Armstrong et al., 2009; Chenevier et al., 2013). As shown for *Escherichia coli*’s hydrogenases for example, these enzymes can be used for enhanced hydrogen production on surface-enlarged nanofiber electrodes (Schlicht et al., 2016). Due to the steep thermal and chemical gradients prevailing in vent environments, enzymes of exceptional stability under various conditions (e.g., temperature or oxygen contents) can be expected to be found. These would be the ideal candidates for biotechnological applications in the field of hydrogen production or energy generation in biofuel cells.

## Conclusion

Hydrogen oxidation, catalyzed by phylogenetically diverse Bacteria and Archaea with versatile metabolic pathways, plays a major role for primary biomass production in chemically distinct deep-sea hydrothermal vent systems. However, the metabolic processes and biogeochemical interactions involved in hydrogen conversion are still not fully understood. Assessing the full hydrogen consumption potential of microbial vent communities has often proved to be difficult as incubation experiments but also metagenomic and metatranscriptomic approaches have their particular limitations: i.e., either in the reproducibility of optimal growth and hydrogen consumption conditions or in the lack of functional proof for the putative hydrogen conversion ability. The development of *in situ* hydrogen consumption measurement techniques that include sampling for subsequent molecular analyses would therefore considerably improve the exploration of hydrogen-converting communities in deep-sea vents. Since the culture-dependent and –independent approaches all exhibit individual limitations in identifying novel mechanisms of hydrogen-based metabolisms, the currently available techniques should ideally be combined to elucidate the full hydrogen utilization potential among the yet uncultured majority.

## Author Contributions

NA and MP wrote the manuscript.

## Conflict of Interest Statement

The authors declare that the research was conducted in the absence of any commercial or financial relationships that could be construed as a potential conflict of interest.
